# Electronic patient self-assessment and management (SAM): a novel framework for cancer survivorship

**DOI:** 10.1186/1472-6947-10-34

**Published:** 2010-06-17

**Authors:** Andrew J Vickers, Talya Salz, Ethan Basch, Matthew R Cooperberg, Peter R Carroll, Foss Tighe, James Eastham, Raymond C Rosen

**Affiliations:** 1 Department of Epidemiology and Biostatistics (AJV, TS, EB) and Department of Surgery (JE), Memorial Sloan-Kettering Cancer Center, New York, USA; 2 Department of Urology (MRC, PRC), University of California, San Francisco, San Francisco, USA; 3 New England Research Institutes, Inc., Watertown, MA, USA

## Abstract

**Background:**

We propose a novel framework for management of cancer survivorship: electronic patient Self-Assessment and Management (SAM). SAM is a framework for transfer of information to and from patients in such a way as to increase both the patient's and the health care provider's understanding of the patient's progress, and to help ensure that patient care follows best practice.

**Methods:**

Patients who participate in the SAM system are contacted by email at regular intervals and asked to complete validated questionnaires online. Patient responses on these questionnaires are then analyzed in order to provide patients with real-time, online information about their progress and to provide them with tailored and standardized medical advice. Patient-level data from the questionnaires are ported in real time to the patient's health care provider to be uploaded to clinic notes. An initial version of SAM has been developed at Memorial Sloan-Kettering Cancer Center (MSKCC) and the University of California, San Francisco (UCSF) for aiding the clinical management of patients after surgery for prostate cancer.

**Results:**

Pilot testing at MSKCC and UCSF suggests that implementation of SAM systems are feasible, with no major problems with compliance (> 70% response rate) or security.

**Conclusion:**

SAM is a conceptually simple framework for passing information to and from patients in such a way as to increase both the patient's and the health care provider's understanding of the patient's progress, and to help ensure that patient care follows best practice.

## Background

In this discussion paper, we propose a novel conceptual framework for management of cancer survivorship: electronic patient Self-Assessment and Management (SAM). SAM was developed in response to what we see as three critical problems in the overall clinical management of cancer survivorship: 1) patients typically do not know how they are doing, 2) health care providers frequently do not know how their patients are doing, 3) patients often do not receive the services that they should.

1. **Patients typically do not know how they are doing**. Fundamental to any patient's relationship with medical care is whether symptoms they are experiencing or test results they receive are "normal", or are unusual in some way and require medical intervention. This is as much a psychological as a medical issue: one of the most well-known benefits of increasingly widespread patient support groups is that patients realize that "I am not the only one going through this"[1]. Patients also want to know what is going to happen to them: "given that I started treatment this many weeks ago, and I am currently feeling like this, what is the chance that I'll start feeling better next month?"

2. **Health care providers typically do not know how their patients are doing**. The stereotypical doctor's advice - "Take two aspirin and call me in the morning" - illustrates a central tenet of medical practice: the doctor assesses the patient's progress and modifies treatment accordingly. There are, however, well known problems in how doctors assess their patients' symptoms. Doctors, and other health professionals, do not consistently assess changes in symptoms systematically, nor do they use questions carefully designed to elicit accurate responses[2]. Clinicians are also subject to various perceptual biases - often underestimating patient pain or dysfunction[3,4]. Accordingly, their perceptions of patient outcomes may diverge, sometimes dramatically, from patients' own reports[5-8].

3. **Patients often do not receive the services that they should**. There is overwhelming evidence that many patients do not receive care that follows accepted clinical practice guidelines. For example, only 50% of Canadian colon cancer patients receive a follow-up colonoscopy within five years of completing treatment[9], while 74% of elderly American breast cancer survivors get overly frequent mammograms[10]. Marked variation in treatment independent of disease risk or other patient factors has been shown to be a pervasive problem in prostate cancer treatment[11-13]. This problem is seen not only in cancer survivorship, nor exclusively in North America, but pervades most medical care settings: a mere 20% of UK patients with severe migraine were taking a triptan[14]; only 35% of US Latinos with diabetes received appropriate eye examinations[15].

All of these problems are exacerbated by fragmentation of care. As a simple example, a patient presents in primary care with urinary symptoms, is referred to secondary care for diagnostic work up, receives surgical treatment for prostate cancer at a tertiary care center, and then is discharged back to the care of the family doctor, who has limited knowledge of prostate cancer. As a result, the patient cannot get feedback from his family doctor as to whether post-operative problems, such as urinary symptoms, are typical or constitute a complication in need of intervention. Similarly, the patient may not be offered appropriate advice on urinary and sexual rehabilitation[16,17], or tests for recurrence.

The Institute of Medicine has acknowledged this fragmentation of care in the context of cancer, noting that after completing cancer treatment, survivors are often lost in the transition between acute and ongoing care[18]. To address this issue, the Institute of Medicine has proposed the development of a survivorship care plan, which summarizes the patient's diagnosis, treatment, and recommended ongoing care. At the end of cancer treatment, all patients should ideally receive a detailed survivorship care plan from their treating physician and then bring it to the first appointment with the primary care physician, in order to facilitate communication between physicians and enhance continuity of care.

### SAM: a proposed solution

SAM marries the idea of a survivorship care plan to modern information technology, providing a comprehensive, informative, and interactive service in real time. The SAM system would incorporate a central database for tracking patient information. At regular intervals, the system would use secure email to contact patients to obtain information on their health. This information would then be analyzed in order to 1) provide patients with information about their progress and how they compare to similar patients, 2) offer generic or tailored medical advice to the patient, 3) create a report for the patient's health care provider that can be added to the clinic notes or electronic medical record. The SAM system would take advantage of information technology to provide patients with individualized, medically relevant, and up-to-date information on disease and lifestyle management in real time.

To illustrate how a SAM system might work, we will use radical prostatectomy as an example. A patient diagnosed with prostate cancer and opting for surgery would be advised by his surgeon to register with the SAM system either through a central website or through the hospital website. The patient would be advised that registering with the SAM system is an important part of post-operative care.

Each patient contact with the SAM system would consist of three stages: data entry, data transfer, and patient self-management. The *data entry *process starts when the patient is sent an email asking him to log on to a central SAM website. The email would be sent by a central database at pre-specified times but would be addressed from the patient's surgeon. The email would contain a link to the secure SAM website, where the patient would log in and enter his data by completing an on-line version of a standardized, validated questionnaire regarding his symptoms. Urinary and erectile dysfunction are the two most common and important side-effects of radical prostatectomy and so the questionnaire would focus primarily on those two symptoms. Patients could log on at any time to report their current health, not only in response to an email invitation. Online recording of patient symptoms has been piloted: models have been developed in which patient self-reports of symptoms, functional status, and health-related quality of life are transmitted to clinicians electronically to prompt discussions or clinical actions: these systems have been well-received by both patients and clinicians[19,20]. Use of electronic data capture minimizes missing or inconsistent data, and there is some evidence that individuals provide more honest answers to computers than in face-to-face interviews, particularly with respect to sensitive information[21].

In the *data transfer *stage, the patient's data would be ported electronically to the patient's clinical medical record, so that the patient's surgeon could assess postoperative recovery directly. The surgeon also could see how his or her patient's results change over time.

The final stage of the SAM is *patient self-management*. Following questionnaire completion, the patient would first be directed to a web page with medical advice specific to the period of follow-up. To return to the example of prostate cancer, there are clinical practice guidelines for follow-up with prostate-specific antigen (PSA) testing and treatment of urinary and sexual dysfunction that are relevant at different periods after treatment. Certain advice would be given to all patients irrespective of their questionnaire responses (such as to get a PSA test); tailored advice may also be given to patients responding in particular ways (such as a patient with long-term urinary dysfunction being advised to consult a urologist). After the presentation of medical advice, the patient would be given various navigation options within the website as follows:

• **Patient self-assessment**. Patients would be able to access a table or graph of their medical progress over time. They would also be to obtain information on their likely prognosis (e.g. "For every 100 men like you who are 6 months after surgery and have completed the questionnaires in the same way, 95 would be fully dry (would not need to use pads) at 12 months").

• **Case history**. Patients would be able to download all, or selected parts, of their own case history (recorded by both their health care provider and themselves) from the SAM system. This could then be transfer to other health providers by email or hard copy (e.g. as a PDF). Each prostate cancer patient's case history might include details of his cancer (stage, grade, PSA levels), treatment (e.g. "open retropubic radical prostatectomy from Dr. Brown at St. Elsewhere's Hospital"), baseline and post-treatment urinary and erectile function scores, details of PSA tests and results, and post-surgery treatment.

• **Patient site**. The patient component of the site would have links to sites with general information on prostate cancer, such as those of the American Cancer Society (http://www.cancer.org) or Prostate Cancer Infolink (http://prostatecancerinfolink.net).

Note that SAM addresses each of the three important needs described in our introduction: patients are provided information on their progress, advice on self-care, and recommendations for the medical care they should be getting. At the same time, health care providers receive information about their patients' progress. In particular, SAM extends the concept of the cancer survivorship care plan by providing recommendations for care in real time at relevant time points. For example, in place of a brochure given on discharge stating that "it is important to get regular postoperative PSA tests", a SAM system would send an email to each patient at the time that he should schedule a PSA test.

### Development of SAM at Memorial Sloan-Kettering Cancer Center

A SAM system is currently under development at Memorial Sloan-Kettering Cancer Center to help manage prostate cancer survivorship.

#### Data entry

**Figure 1:**
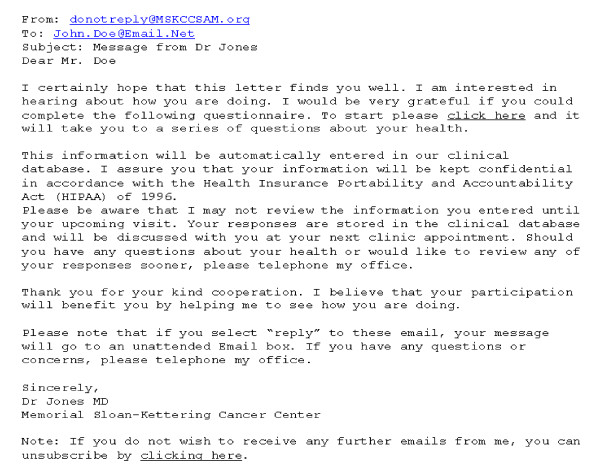
**Example reminder Email to patient to complete MSKCC online questionnaire**.

**Figure 2:**
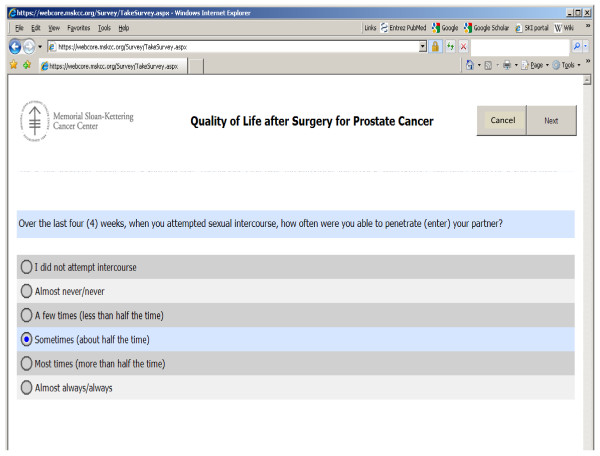
**Screen shot from MSKCC online questionnaire**.

We have developed an online questionnaire to assess patient recovery after radical prostatectomy. Patients are sent an email by their surgeon every 3 months after surgery for the first year, at 18 months, 2 years and then yearly afterwards. The text of the email is shown in figure 1. Patients click on a hyperlink in the email to reach a log-in page and then complete an online questionnaire. This includes the International Index of Erectile Dysfunction [22], 6 questions on urinary function and 2 questions on bowel function taken from the Memorial instrument for health-related quality of life in prostate cancer[23], and a 0 - 10 numerical rating scale of health related quality of life. A screen shot from the questionnaire is shown in figure 2. The questionnaire includes interactive branching logic. For example, a man reporting that he is not sexually active is not asked further questions about erectile function; the first time that a man reports that he is not in need of pads to control urinary leakage he is asked when he stopped using pads.

At the time of writing, 1795 MSKCC patients are registered to the SAM system, of whom 1052 have received at least one questionnaire. Approximately 75% of questionnaires are completed by patients within two weeks of the initial email. Some patients have failed to complete a baseline questionnaire because they were unable to log in to the website; accordingly we have changed our log in procedure and expect the compliance rate to increase.

#### Data transfer

**Figure 3:**
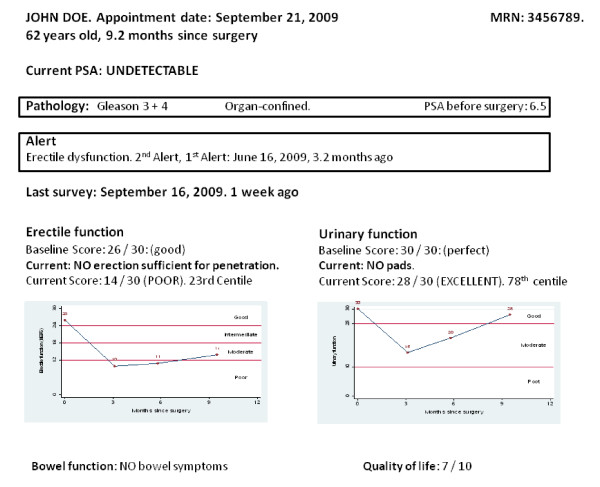
**Example report used by surgeon during clinic visit**. The data are for a hypothetical patient.

Data from the questionnaire are transferred automatically to a clinical database, where a summary report can be accessed by the surgeon prior to the patient's subsequent follow-up clinic visit. The database report currently contains an overview of the patient's latest questionnaire responses, but we are currently developing a more advanced report that illustrates patient progress over time and provides "alerts" of symptoms that might require further follow-up or intervention (figure 3).

#### Patient self-management

**Figure 4:**
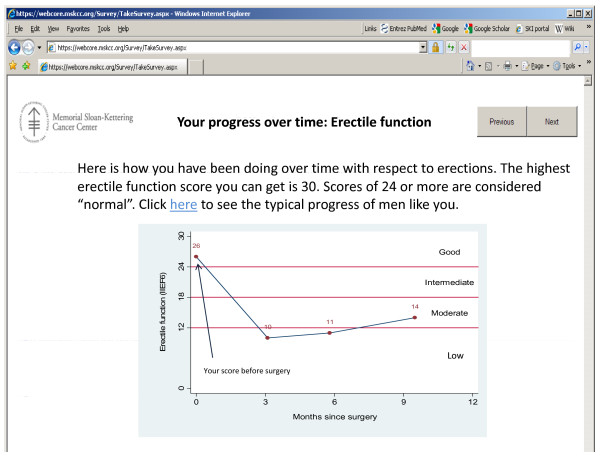
**Template for patient self-management portal: review of progress over time**.

**Figure 5:**
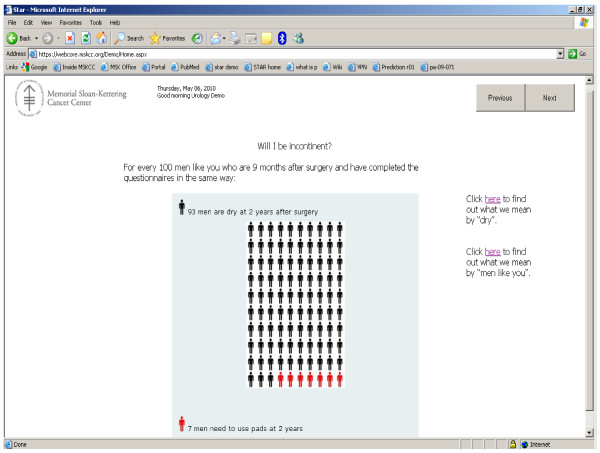
**Template for patient self-management portal: predicted outcomes**.

At the time of writing, code for the patient self-management aspect of SAM is under development, and has yet to be implemented at MSKCC. Figures 4 and 5 show templates for the patient progress and predictions. The template for advice to be given to patients is given as additional file 1.

### Development of SAM at the University of California, San Francisco

Over the past two years, the Urology Oncology and Breast Oncology groups at the UCSF Helen Diller Family Comprehensive Cancer Center have worked with Dynamic Clinical Systems (DCS, Hanover, NH) to develop a pilot SAM system for patients with prostate and breast cancer.

#### Data entry

Prior to their initial visit, new patients complete a full health history questionnaire via a secure on-line portal, and also report their baseline general and disease-specific health-related quality of life. Surveys for men with prostate cancer include the International Prostate Symptom Scale (IPSS), the Expanded Prostate cancer Index Composite-26 (EPIC),[24] and the Sexual Health Inventory for Men, as well as the SF-12 and standard depression and anxiety screening tools. Patients complete a shorter version of the questionnaire, including updates of the quality-of-life assessments, with each return visit. Those who have travelled to UCSF for consultation or surgery but will be followed locally by other clinicians after initial management, receive the surveys on a fixed interval rather than in association with clinic visits.

To date, 1713 men have been registered in the system, and 4566 surveys have been scheduled. Of these, 2313 (51%) have been completed, and another 224 (5%) partially completed. The completion rate was higher for initial surveys (72%) than for followup surveys (44%). In only 4 cases did patients expressly refuse to complete the survey--the remainder of the incomplete surveys reflect a variety of factors reflecting a technology in evolution, including problems with email addresses and browser compatibility as well as challenges in integrating the software with existing clinic software and processes.

Patient-reported data are merged with clinical data within UCSF's Urologic Oncology Data Base, and are also used to generate patient summaries for use in the clinic setting, which, similar to the MSKCC system, includse graphing functions and highlighting of "red flags" related to HRQOL progress and other aspects of pre- and post-treatment care. As with the MSKCC system, patient-oriented reports, including lay language interpretation of findings and benchmarking of outcomes to those of men in CaPSURE with similar disease characteristics, are in development.

### Novel aspects of SAM

Several features of SAM have been developed in the context of patient self-management programs for chronic diseases, in which patients use the internet or text messaging to report symptoms and quality of life. Ralston et al. piloted a web-based patient self-assessment tool that elicited symptom reports and blood glucose levels from diabetic patients[25]. Similarly, two asthma self-management programs ask patients to report their peak expiratory flow and forced expiratory volume over the internet or as a text message[26,27]. A program developed by Nguyen et al. for self-management of chronic obstructive pulmonary disease requires patients to report their symptoms and their exercise routines[28]. Each of these programs contains components of the SAM system.

With respect to cancer, two programs have used the internet to elicit information about symptoms and quality of life from patients[29-31]. These self-management programs varied in how patient-reported information was collected and used. Patients may receive automatically generated advice about disease management in response to self-reported symptoms,[30] or information about how the patient's self-reported evaluations compare to earlier evaluations or symptom reports[26,27]. Some programs provided patient-reported information to healthcare providers,[28,29,31] and in other programs, the information was integrated directly into the electronic medical record[25,27].

Recent years have also seen the development of online advice for patients via public web sites. One example is Oncolife, which is part of Oncolink, a well-known website providing information about cancer. In brief, patients complete a short survey about their age, diagnosis and treatment and then receive a survivorship care plan specific to their particular needs.

What is novel about the SAM concept is that it ties key elements of these various approaches together into a coherent system for how information technology can enhance management of chronic diseases like cancer. In place of systems that either plot a patient's symptoms over time[27] or provide information and advice,[28,30] SAM does both, using patient symptoms to guide information and advice. In place of systems that feed patient-reported outcomes to either the physician's case notes,[25,29] or a patient-held record,[26,30] SAM allows patients access to their own records and reports while also transferring data to their physician. In place of a static survivorship care plan, SAM would include longitudinal follow-up, email reminders ("It has been one year since surgery, you should have a PSA test" instead of being told at baseline that "A PSA test is recommended one year after surgery"), and modification of the care plan depending on patient symptoms and cancer health state.

### Evaluation of SAM

The complete SAM system has yet to be implemented and, as such, SAM has not yet been evaluated for usability and effectiveness. However, when it becomes available, we plan several systematic evaluations of SAM. We will collect patient data on usability, satisfaction with the system, and satisfaction with medical care. We will also assess physician satisfaction and determine any effects of SAM on resource use. There are several possible methodologies for such a study. For example, patients currently completing the questionnaires online could be offered participation in a trial, in which they would be randomly assigned to get additional information (plots of their symptoms over time, advice) or not. Alternatively, routine satisfaction with care questionnaires could be compared before and after the full implementation of SAM, on the grounds that strong secular trends in satisfaction would be unlikely over a short period of time.

## Conclusions

Medical use of the Internet has emphasized flat, one-way passage of information: a typical website dealing with recovery after cancer surgery, for example, provides little more than an on-line brochure. To date, there have been remarkably few attempts to make use of information technology for interactivity in real-time, personalized information, longitudinal follow-up, or data analysis.

In this paper, we have proposed a conceptually simple framework for passing information to and from patients in such a way as to increase both the patient's and the health care provider's understanding of the patient's progress, and to help ensure that patient care follows best practice. This system has been implemented in part at Memorial Sloan-Kettering Cancer Center and UCSF, demonstrating feasibility.

## List of abbreviations

PSA: prostate specific antigen; SAM: Electronic patient self-assessment and management.

## Competing interests

The authors declare that they have no competing interests.

## Authors' contributions

AJV conceived the idea behind the paper and wrote the first draft. TS contributed to the original idea and rewrote the manuscript to focus on survivorship. EB advised on the use of patient-reported outcomes. MRC and PRC contributed the experienced of the UCSF site; JAE helped lead the MSKCC piloting of SAM. FT and RCR contributed from a medical informatics standpoint. All authors revised the manuscript critically for important intellectual content and approved the final manuscript.

## Pre-publication history

The pre-publication history for this paper can be accessed here:

http://www.biomedcentral.com/1472-6947/10/34/prepub

## Supplementary Material

Additional file 1 **Patient advice template**. Shows the text provided to patients given particular responses to the survey. Click here for file
