# Supplementary material for: Electronic patient self-assessment and management (SAM): a novel framework for cancer survivorship
Source: BMC Med Inform Decis Mak. 2010 Jun 17;10:34. doi: 10.1186/1472-6947-10-34 (PMC2893444; doi:10.1186/1472-6947-10-34)
Supplement: Additional file 1 — Patient advice template. Shows the text provided to patients given particular responses to the survey. [file 1472-6947-10-34-S1.PDF]

Message to patients	Criteria
Generic	
Also, continue doing your kegel exercises. If you don't know how to do these, call your doctor's office for further information.	All patients at 3 months
Remember that recovering sexual function takes time. The goal is to get you to a functional level of sexual ability, if this is important to you. Continue to be sexually active if that is what you want. Several sexual problems are common after prostate cancer surgery, including:<br>1. Changes in the shape of your penis, such as curving to the left or right<br>2. Pain during orgasm<br>3. Leaking urine during orgasm<br>If you are experiencing any of these, you should considering speaking with your urologist regarding treatments. If you are leaking urine during sex, you can also try emptying your bladder just before having sex, and limiting fluids a few hours prior to sexual activity.	All patients at time-points up to 2 years.
If you haven't already done so, it is time to schedule an appointment with your doctor for a PSA test.	All patients at all follow-up times.
Urinary	
Your responses to the survey indicate you are having difficulty with urination Many patients after surgery for prostate cancer don't have control of urine. However, most patients get better over time and it is unusual to still have problems with urine a year or more after surgery. You should consider discussing your problems with your doctor. There are treatments available that might be able to help you.<br><br>There are some other things that you can try to help deal with urinary problems. You can restrict the amount of fluid you drink. You can also limit or avoid caffeine. Caffeine can be very irritating to the bladder causing increase in leakage. You can use protective pads and empty your bladder before drinking alcohol, exercising, or engaging in any activity that would make it difficult to use the bathroom.	Patients reporting that urinary function is moderate or big problem at any point at 12 months or after<br>OR<br>Use of pads at 12 months or after
Remember to keep up with your kegel exercises daily.<br><br>There are some other things that you can try to help deal with urinary problems. You can restrict the amount of fluid you drink. You can also limit or avoid caffeine. Caffeine can be very irritating to the bladder causing increase in leakage. You can use protective pads and empty your bladder before drinking alcohol, exercising, or engaging in any activity that would make it difficult to use the bathroom.	Patients reporting that urinary function is moderate or big problem at 6 or 9 months<br>OR<br>Use of pads at 6 or 9 months

Message to patients	Criteria
Your responses to the survey suggest that your urinary function has been declining over time. In some cases, this may indicate a problem that can be treated. See your urologist or a voiding dysfunction specialist to help you with this problem.	Patients with any worsening in score at 6,9, and 12 months<br><br>OR<br><br>Patients reporting worsening of score by at least 3 points compared to any previous point at 18+ months
Sexual	
Your responses to the survey indicate that you are having some difficulty with sexual function.<br><br>To help you with any difficulty getting erections, talk to your urologist about therapies you can try, such as medication (like Viagra, Levitra, or Cialis). If you are already taking medication, talk to your doctor or nurse practitioner about other options, such as penile injection therapy, urethral suppository, or a vacuum device. Remember that it can take up to two years for the nerves that control erections, the cavernous nerves, to recover from the trauma of surgery.	Patients reporting IIEF score <24 at any point
Bowel	
You indicated that you are having difficulty with your bowels. This is not common after prostate cancer surgery and can indicate a problem. It is important to discuss this problem with your surgeon.	Patients reporting bowel habits are moderate or big problem at any point<br><br>OR<br><br>Patients reporting bowel problems made it difficult to enjoy life frequently or most of the time at any point
